# Rhubarb Enema Decreases Circulating Trimethylamine N-Oxide Level and Improves Renal Fibrosis Accompanied With Gut Microbiota Change in Chronic Kidney Disease Rats

**DOI:** 10.3389/fphar.2021.780924

**Published:** 2021-12-13

**Authors:** Chunlan Ji, Yin Li, Yenan Mo, Zhaoyu Lu, Fuhua Lu, Qizhan Lin, Xusheng Liu, Chuan Zou, Yuchi Wu

**Affiliations:** ^1^ The Second Clinical Medical College, Guangzhou University of Chinese Medicine, Guangzhou, China; ^2^ Department of Nephrology, Guangdong Provincial Hospital of Chinese Medicine, Guangzhou, China; ^3^ State Key Laboratory of Dampness Syndrome of Chinese Medicine, The Second Affiliated Hospital of Guangzhou University of Chinese Medicine, Guangzhou, China

**Keywords:** rhubarb, trimethylamine N-oxide, chronic kidney disease, renal fibrosis, gut microbiota

## Abstract

**Objectives:** Trimethylamine N-oxide (TMAO), a metabolic product of gut flora, is increased in chronic kidney disease (CKD) subjects and is recognized as one type of uremic toxins which is associated with poor cardiovascular outcomes and kidney function loss. Previous studies have suggested that rhubarb enema could reduce circulating uremic toxins such as urea, creatinine, and indoxyl sulfate and also regulate the intestinal microbiota. However, whether rhubarb enema retards kidney dysfunction by reducing circulating TMAO and its underlying mechanism, are still unclear. The present study aims to investigate the impact of rhubarb enema on TMAO and its precursors, as well as on the intestinal microbiota in 5/6 nephrectomized (5/6Nx) CKD rats.

**Design**: Rats in the treatment groups were given rhubarb enema after modeling. At the end of the study, blood, feces, and kidney tissues were collected and processed for biochemical analyses, histological and western blot analyses, 16S rRNA sequence and untargeted metabolomic analyses.

**Results:** Rhubarb enema reduced serum TMAO and trimethylamine (TMA) levels, inhibited the expression of inflammatory markers (interleukin-6, tumor necrosis factor *α* and Interferon-*γ*) and alleviated tubular atrophy, monocyte infiltration and interstitial fibrosis in 5/6Nx CKD rats. Moreover, rhubarb enema significantly increased the abundance of some symbiotic bacteria and probiotics*,* while reduced the abundance of some potential pathogens at the genus level. In addition, Spearman’s correlation analysis revealed that *lachnospiraceae and romboutsia* were positively correlated with TMAO.

**Conclusion:** Rhubarb enema decreases circulating TMAO level and improves renal fibrosis in 5/6Nx CKD rats, which may be related to the regulation of intestinal microbial community.

## 1 Introduction

More than 850 million individuals have kidney diseases around the world (Jager et al., 2019). With the increasing incidence of diabetes and cardiovascular and cerebrovascular diseases, the number of people suffering from chronic kidney disease (CKD) is expected to increase and result in elevated economic burden of medical expenditures (Crews et al., 2019). Therefore, the international nephrology community has been exploring the pathogenesis, the prevention and treatment strategies of CKD.

In recent years, some scholars have proposed the gut-kidney axis theory, that is, the intestine and the kidney correlate to each other closely (Stavropoulou et al., 2021). There are huge intestinal microbes in the human intestine, up to 3.9*10^13^. Bacteria outnumber human cells by a ratio of at least 10:1 (Sender et al., 2016), keeping symbiosis with the host. The abundance and structure of intestinal microbes are affected by factors such as diet, environment, and diseases (Sidhu and Poorten, 2017). In CKD environment, the abundance and structure of intestinal microbes change significantly (Meijers et al., 2019; Ren et al., 2020), resulting in changes in the metabolites of the flora, such as increased production of ammonia, urea, indoxyl sulfate (IS), trimethylamine-N-oxide (TMAO), etc. Meanwhile, due to impaired kidney function, the excretion of these flora products is decreased, contributing to the accumulation of uremic toxins in the body (Ramezani et al., 2016). Excessive circulating uremic toxins lead to inflammatory damage and fibrosis of the kidneys (Zeisel and Warrier, 2016). As the disease worsens, the flora is further affected, and the harmful metabolites further accumulate, thus forming a cycle of evil.

Gut microbes, primarily those from the families *Clostridia* and Enterobacteriaceae, participate in the degradation of nutrients such as carnitine, choline and betaine, forming trimethylamine (TMA) (Rath et al., 2017). Then, TMA is oxidized into TMAO by the flavin containing monooxygenase isoform three enzyme (FMO3) in the liver. Circulating TMAO levels are low in healthy subjects but markedly increased in CKD patients, which presents a strong inverse association with residual renal function (Posada-Ayala et al., 2014; Stubbs et al., 2016). TMAO can not only induce endothelial dysfunction, vascular atherosclerosis, but also aggravate renal fibrosis (Zeisel and Warrier, 2016; Al-Obaide et al., 2017; Ma et al., 2017).

Targeting the intestinal-renal axis is deemed promising and important for the treatment of CKD. Enema, literally a treatment for cleaning the bowels by filling them with a liquid through the anus, has been adopted to treat diverse diseases. Using Chinese herbal liquid for enema was originally recorded in “Treatise on Febrile Diseases” in the third century AD and continuously applied in clinical practice till today. For chronic renal failure, especially end-stage renal disease, enema therapy using Traditional Chinese medicine (TCM) compound containing rhubarb has been found by clinical observations to have significant effect in reducing uremic toxin accumulation, preserving kidney function and improving systemic inflammation (Zou et al., 2012; Zou et al., 2013). Previously we found that rhubarb enema treatment modified the diversity of gut microbiota in CKD rats, inhibiting the overgrowth of conditional pathogenic gut bacteria such as *Akkermansia, Methanosphaera, and* Clostridiaceae. Other gut bacteria like *Clostridium, Alistipes,* one member from family Clostridiaceae, and one member from family Enterobacteriaceae have more abundance after intervention (Lu et al., 2015; Ji et al., 2020). However, whether the modification to gut flora by rhubarb enema affects TMA/TMAO metabolism and circulating TMAO level, and what is the underlying mechanism, are still unclear. Thus, this study intends to investigate whether rhubarb enema for CKD rats induce change of the intestinal microbes, thereby reducing serum TMAO level, improving systemic inflammation levels, and ultimately alleviating renal fibrosis.

## 2 Materials and Methods

### 2.1 Animals and Models

40 male specific pathogen-free (SPF)-grade Sprague-Dawley rats weighing about 220 ± 20 g were purchased from Experimental Animal Center of Southern Medical University and housed in the SPF breeding room of Guangdong Academy of Chinese Medicine [SCXK (Yue) 2013-0002, Guangzhou, China].

The classic 5/6 nephrectomy (Nx) model was used in this study. After 3 days of quarantine and 7 days of adaptive feeding, 30 of the rats were modeled with 5/6 nephrectomy to simulate the state of CKD. After 4 weeks of healing and stabilization, blood samples were collected for baseline serum creatinine. Rats were randomly divided into three groups with baseline creatine equilibrium: 5/6Nx model group, rhubarb enema low-dose group (rhubarb L group) and rhubarb enema high-dose group (rhubarb H group). Another 10 sham-operated rats were used as the control group. This animal experiment has been approved by the Animal Ethics Committee of Guangdong Provincial Hospital of Chinese Medicine (No. 2019028).

### 2.2 Preparation of Enema Drugs

Rhubarb granules were purchased from Jiangyin Tianjiang Pharmaceutical (Jiangsu, China). 1 g of rhubarb granules is equivalent to 3 g of raw rhubarb. In clinical practice, 30 g of raw rhubarb (i.e. 10 g of rhubarb granules) were decocted into 100 ml enema solution for a patient weighing around 60 kg. So, the dosage of rhubarb granules for clinical subjects was 0.167 g/kg. According to the body surface area calculation method, the weight-averaged dosage for rats was about 6.3 times that for humans. Thus, the dose of rhubarb granules administered for rats were 1.05 g/kg for the low dose group, and 2.10 g/kg for the high dose group. For the experiment, 10 and 20 g of rhubarb granules were dissolved and mixed well in 100 ml of deionized water respectively, making rhubarb enema solutions with low and high concentrations (0.1 g/ml and 0.2 g/ml). They were stored in a sealed container and heated to 37°C before use. The volume of rhubarb enema solution for each rat was calculated based on the required dosage of rhubarb and the concentration of rhubarb solution.

### 2.3 Method of Enema

The rats were fasted for 12 h before enema. Each rat was held on the rat cage at the head and neck so that the tail and the anus were presented in front, exposing the anus. The abdomen of the rat was kneaded to promote defecation. A straight-head gavage needle was slowly inserted into the rat rectum about 6 cm deep after it was lubricated with glycerin. The gavage needle was held in the fixed position and about 5 ml (individually calculated) of rhubarb solution was slowly injected using a sterile syringe. After that, the gavage needle was pulled out, and the anus was pinched for 2 min to achieve a better retention effect. Enema were given once a day for 8 weeks.

### 2.4 Biochemical Testing

24-h urine was collected using a metabolic cage, and sent to Laboratory Department of Guangdong Provincial Hospital of Chinese Medicine for urine protein and urine creatinine detection.

After 8-weeks rhubarb enema, the rats were sacrificed and had their blood samples taken from the abdominal aorta. Serum samples were sent to the Laboratory Department as mentioned above. Serum creatinine and blood urea nitrogen were measured using an automatic biochemical analyzer (Hitachi, 7180, Tokyo, Japan).

### 2.5 H&E Staining

Rat kidneys tissues were taken and immersed in 10% formalin solution for 36 h, then were put into an automatic dehydrator (Leica, ASP300S, Munich, Germany) for dehydration, and embedded in paraffin (Leica, Munich, Germany). Paraffin specimen of kidney was cut into 3 μm. After dewaxing with xylene, kidney paraffin sections were immersed in gradient alcohol and water before staining. Hematoxylin stain for 5 min and eosin stain for 10 min were performed, following by dehydration with gradient alcohol and hyalinization with xylene. Then kidney sections were mounted with neutral gum. Finally, histological images were captured using a microscope (Hitachi). The H&E kit was purchased from Beyotime Biotechnology (Cat. No. C0105S, Shanghai, China).

### 2.6 Immunohistochemical Staining

After dewaxing with xylene, kidney paraffin sections were immersed in gradient alcohol and water before staining, then soaked in 3% H_2_O_2_ to remove endogenous peroxidase. All sections were heated in sodium citrate solution for 15 min in 98°C for antigen retrieval. Then the tissue specimens were circled with an immunohistochemical pen, added 5% bovine serum albumin (BSA) solution, and blocked at 37°C for 30 min, incubated with the primary antibodies including fibronectin (1:500, abcam, ab268021, United Kingdom, Cambridge), alpha smooth muscle actin (1:300, abcam, ab7817, United Kingdom Cambridge), collegen-I (1:500, abcam, ab270993, United Kingdom, Cambridge) solution 200 μL at 4°C overnight. After washing off, these tissues were incubated with secondary antibody solution at 37°C for 40 min, colored by 3,3′-diaminobenzidine (DAB) under a microscope and counterstained with hematoxylin (Cat. No. C0105S, Beyotime Biotechnology, Shanghai, China). The immunohistochemistry kits were purchased from Boster Biotechnology (Fujian, China). DAB color reagent kits were purchased from Maixin Biotechnology (Fujian, China).

### 2.7 TMAO Targeted Metabolomics

Targeted metabolomics is the measurement of defined groups of chemically characterized and biochemically annotated metabolites. Through the use of internal standards, analysis can be undertaken in a quantitative manner. When utilizing targeted metabolomics sample preparation can be optimized, reducing the dominance of high-abundance molecules in the analyses. In addition, since all analyzed species are clearly defined, analytical artifacts are not carried through to downstream analysis.

Multi-reaction monitoring technology (MRM) using standard products as a reference, performs targeted and specific detection and analysis of specific metabolite groups, and can obtain absolute quantitative results of target metabolites, with features of strong specificity, high sensitivity and high accuracy. Based on known or assumed reactive ion information, MRM technology selectively selects data for mass spectrometry signal acquisition, records the signals of ion pairs that conform to the rules, and removes the interference of non-compliant ion signals. In the process of quantitative analysis, the technology first screens the target metabolite-specific precursor ions, and then selectively induces collisions with these precursor ions, removes interference from other ions, and only collects mass spectrometry signals on selected MS/MS2 ions so as to achieve a more specific, sensitive and accurate analysis of the target metabolic molecule (Roberts et al., 2012).

The separation was performed on a UPLC system (Agilent 1290 Infinity UHPLC) on a HILIC column (Waters, BEH HILIC 2.5 µm, 2.1 mm × 100 mm column) by gradient elution. Eluent A was acetonitrile, and Eluent B was water consisting of 10 mM ammonium formate buffer (pH 3.5). The gradient elution program was as followed: 0 min = 90% B, 1.5 min = 90% B, 4.5 min = 87% B, 7 min = 85% B and 7.5 min = 50% B, and 10 min = 50% B, 10.5 min = 90% B and 14 min = 90% B. Before injecting the next sample, the column was equilibrated with the initial mobile phase for 5 min. The flow rate was constant at 0.4 ml/min and the column temperature was set at 25°C. 5500 QTRAP (AB SCIEX) was performed in positive switch mode. The ESI source conditions were as followed: source temperature: 550°C; ion Source Gas1 (Gas1): 55; Ion Source Gas2 (Gas2): 55; Curtain gas (CUR): 40; ion Sapary Voltage Floating (ISVF): +4500 V. MRM method was used for mass spectrometry quantitative data acquisition. Data analysis MultiQuant or Analyst was used for quantitative data processing. Standard curves and MRM parameters were presented in Table 1 and Table 2.

**TABLE 1:** Standard curves for target compounds.

No	Component name	Mass info	Retention time	Linear	R
1	Betaine	118.1/58.1	5.089,299,063	y = 0.00533 x + 0.03247	0.99975
2	Creatinine	114.1/44.0	2.007,970,744	y = 0.00554 x + 0.02928	0.99970
3	TMA	60.1/44.1	2.629,976,991	y = 0.00418 x + 0.05145	0.99968
4	TMAO	76.1/58.0	2.405,681,062	y = 0.01183 x + 0.57592	0.99907
5	Carnitine	162.1/103.1	3.30,110,464	y = 0.00695 x + 0.02318	0.99983
6	Choline	104.0/60.1	3.042,922,928	y = 0.00416 x + 0.02534	0.99907

**TABLE 2:** Target compounds Ratio of electric charge to mass and retention time.

Metabolite name	Transitions	Retention Time (min)
TMA	60.1/44.1	2.629,976,991
TMAO	76.1/58.0	2.405,681,062
Betaine	118.1/58.1	5.089,299,063
Creatinine	114.1/44	2.007,970,744
Carnitine	162.1/103.1	3.30,110,464
Choline	104/60.1	3.042,922,928

### 2.8 Enzyme-Linked Immunosorbent Assay

The levels of serum interleukin-6 (IL-6), tumor necrosis factor *α* (TNF-α) and Interferon-*γ* (IFN-*γ*) were detected using ELISAs kits. IL-6 (CSB-E04640r), IFN-*γ* assay kit (CSB-E04579r) and TNF-α (CSB-E04640r) assay kits were obtained from Wuhan Huamei Bioengineering Co., Ltd. (Wuhan, China).

### 2.9 Sequencing and Bioinformatics Analysis of Intestinal Flora

Fresh feces of rats were collected using sterile forceps in a position described in Section 2.3 Method of enema. Fecal samples were placed into Eppendorf (EP) tubes, and stored at −80°C.

DNA samples were quantified using a NucleoSpin Soil Kit-Macherey-Nagel (Biocompare, Germany) and then transferred to Beijing Genomics Institute (BGI) Group for gene sequencing of the V4 region of the 16S rRNA gene with the Hiseq 2500 (Illumina, California, United States). PCR primers used for the 16S rRNA amplicon libraries were 515F and 806 R.

Clean reads were obtained from the raw sequencing data by eliminating adapter contamination and low-quality data. Overlapping paired-end reads were merged to tags and then clustered to operational taxonomic units (OTUs) at 97% sequence similarity using FLASH (version 1.2.11) and USEARCH (version 7.0.1090). OTU representative sequences were sorted by taxonomic ranks based on the Ribosomal database Project Classifier (version 2.2) trained on the database Greengene_2013_5_99, setting the cutoff confidence values as 0.6. Predictive functional classification schemes of KEGG Orthology were performed using PICRUSt (version 1.1.3). R software (version 3.5.0; R Foundation for Statistical Computing, Vienna, Austria) were used for all statistical analyses. Alpha diversity analysis (Shannon index) and principal coordinate analysis (PCoA) based on Bray-Curtis dissimilarity matrices were performed at the genus level for 16S rDNA gene sequencing using the vegan package. Alpha diversity among groups were compared using Kruskal-Wallis test. Permutational multivariate analysis of variance (PERMANOVA) was performed on dissimilarity matrices to assess the effects of groups with 10,000 permutations in R (version 3.5.0, vegan package). Linear discriminant analysis of effect size (LEfSe) was applied to identify features that differed significantly among groups, as well as their effect sizes. EnvFit analysis was used to determine the effect size and significance of each covariate among the centroids of each group with 999 permutations using the envfit function; redundancy analysis (RDA) was performed using the rda command of the vegan package. Spearman Rank Correlation was applied to investigate associations between differential microbiota/KO and environmental variables; the false discovery rate was used to assess the significance of differences with *p* < 0.05.

### 2.10 Statistical Methods

SPSS software (version 23.0, IBM Corporation, Almonk, New York, United States) was used for statistical analysis. Normally distributed data were presented as mean ± standard error. Independent samples test was used to compare the data with homogeneous variance between two groups of normal distribution. For multiple sets of data, single factor analysis of variance was applied. Non-parametric Mann-Whitney test was used to compare non-normally distributed data with heterogeneous variance between the two groups. The non-parametric rank sum test was used to compare rank data between groups. A *p* value < 0.05 was considered statistically significant.

## 3 Results

### 3.1 General Animal Conditions and Biochemical Analysis Results

There was no statistical difference in the body weight of rats among the four groups. The residual kidney was compensatory hyperplasia after modeling. Compared with the control group, the total amount of residual kidney in the 5/6Nx model group was significantly larger (*p* = 0.0023). The weight of residual kidney in the rhubarb L group and the rhubarb H group had no statistical difference compared with the 5/6Nx model group (*p* = 0.5596, *p* = 0.9818).

The baseline creatinine of the 5/6Nx model group was significantly higher than that of the sham group (*p*<0.001), while there was no statistical difference between the rhubarb L group and the rhubarb H group (*p* = 0.4789, *p* = 0.9749). After rhubarb enema, the creatinine level of the model group was significantly higher than that of the sham group (*p*<0.001), while the creatinine levels of the rhubarb L group and the rhubarb H group were significantly lower than that of the 5/6Nx model group (*p* = 0.0131, *p =* 0.0041). Compared with the 5/6Nx model group, high-dose of enema rhubarb could significantly reduce serum urea level (*p*<0.001).

After modeling, the urine output of rats significantly increased, compared with the sham group (*p*<0.001). Rhubarb enema intervention reduced the urine output, compared with the 5/6Nx model group, which was of statistical significance for the rhubarb L group (*p* = 0.0241). In terms of urine creatinine and urine protein concentration, there were no significant differences among the four groups (*p* = 0.7142, *p* = 0.0736). The urine protein-creatinine ratio of the 5/6Nx model group was significantly higher than that of the sham group (*p* = 0.0055), while the rhubarb L group and the rhubarb H group had no statistical difference compared with the 5/6Nx model group (*p* = 0.1042, *p* = 0.3033) (Table 3).

**TABLE 3:** General conditions and biochemical analysis.

Parameters	Sham (*n* = 8)	5/6Nx (*n* = 8)	5/6Nx + rhubarb L (*n* = 8)	5/6Nx + rhubarb H (*n* = 8)
Body weight (g)	543.88 ± 86.82	540.13 ± 60.09	530.75 ± 28.62	534.25 ± 59.83
Residual kidney weight (mg)	1361.38 ± 140.81	1858.13 ± 355.82^*^	1977.63 ± 264.75	1805.13 ± 235.24
Residual kidney weight/Body weight (mg/g)	2.50 ± 0.13	3.47 ± 0.75	3.73 ± 0.47	3.38 ± 0.19
Serum Creatinine (baseline, μmol/L)	24.40 ± 3.51	72.75 ± 13.94^*^	68.38 ± 9.75	72.50 ± 17.08
Serum Creatinine (The end, μmol/L)	67.30 ± 10.18	120.27 ± 13.79^*^	101.81 ± 12.21^#^	97.95 ± 11.93^#^
Urea (mmol/L)	4.57 ± 0.39	8.84 ± 1.84^*^	8.12 ± 0.90	5.33 ± 0.83^#^
Urine volume (ml)	13.50 ± 2.45	29.63 ± 5.26^*^	23.75 ± 4.71^#^	27.50 ± 7.89
Urine Creatinine (μmol/L)	12,569.50 ± 4,240.85	6,425.63 ± 2917.77^*^	9,228.75 ± 513.27^#^	6,688.63 ± 1710.85
Urine protein concentration (mg/L)	811.00 ± 466.83	993.88 ± 820.56	1282.25 ± 343.22	921.25 ± 805.56
UPCR (g/g)	0.54 ± 0.13	0.84 ± 0.22^*^	1.23 ± 0.34	1.23 ± 1.18

Abbreviations: UPCR, urine protein creatinine ratio. 5/6Nx, 5/6 nephrectomy. 5/6Nx + rhubarb L, 5/6 nephrectomy + low dose rhubarb enema. 5/6Nx + rhubarb H, 5/6 nephrectomy + high dose rhubarb enema. * vs sham, # vs model.

### 3.2 H&E Pathological Staining

**FIGURE 1:**
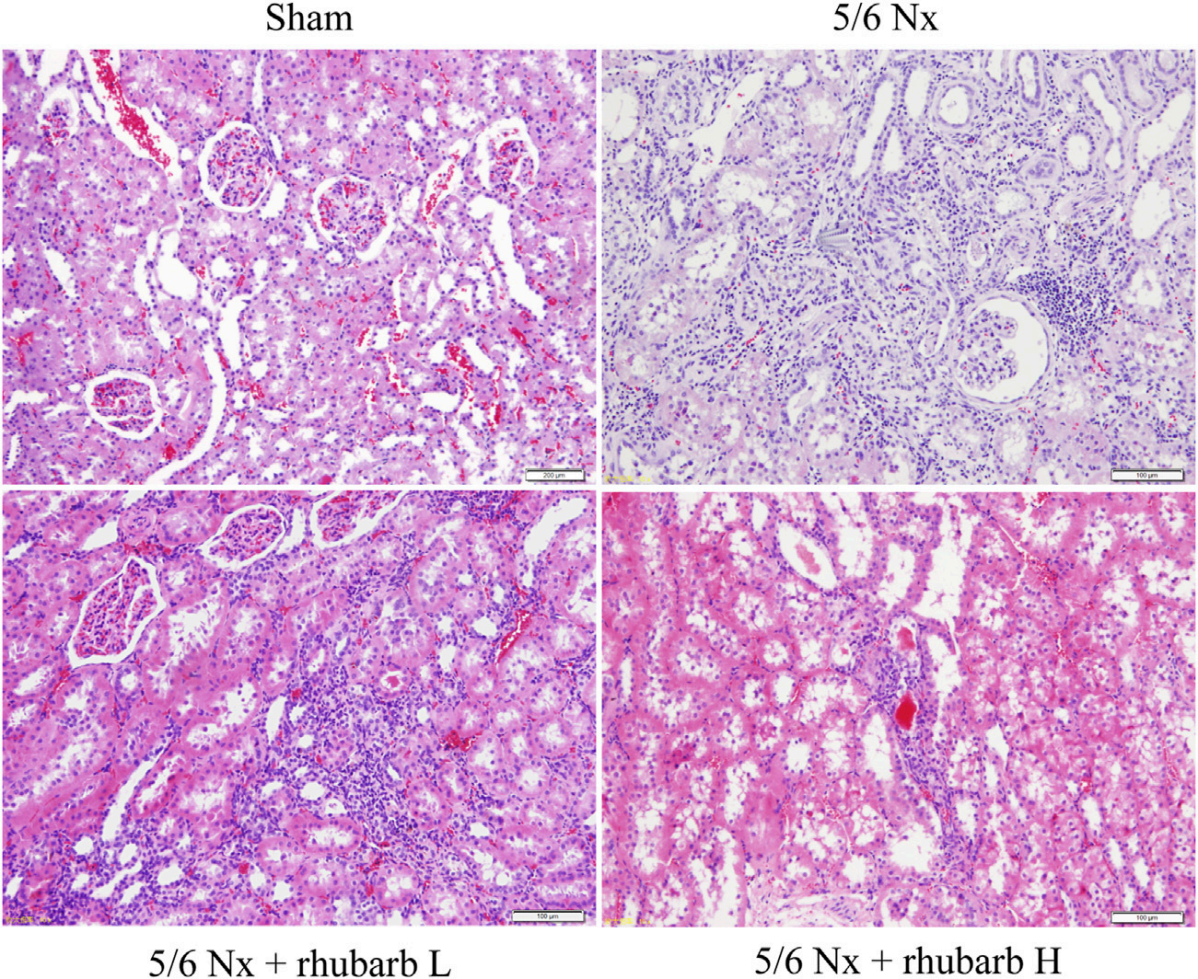
Change of Kidney pathology. H and E staining. In 5/6Nx rats, a large number of inflammatory cells infiltrated the kidney tissue, and interstitial fibrosis and renal tubular atrophy occurred. Rhubarb enema could alleviate these pathological changes (200×).

Compared with the control group, the renal tubules in the 5/6Nx model group were apparently atrophic. Lymphatic mononuclear cells infiltration and renal interstitial fibrosis were present in the 5/6Nx model group. After rhubarb enema intervention, renal tubule atrophy, monocyte infiltration and renal interstitial fibrosis reduced, compared with the 5/6Nx model group, which was better improved in the rhubarb H group than in the rhubarb L group (Figure 1).

### 3.3 Immunohistochemistry

**FIGURE 2:**
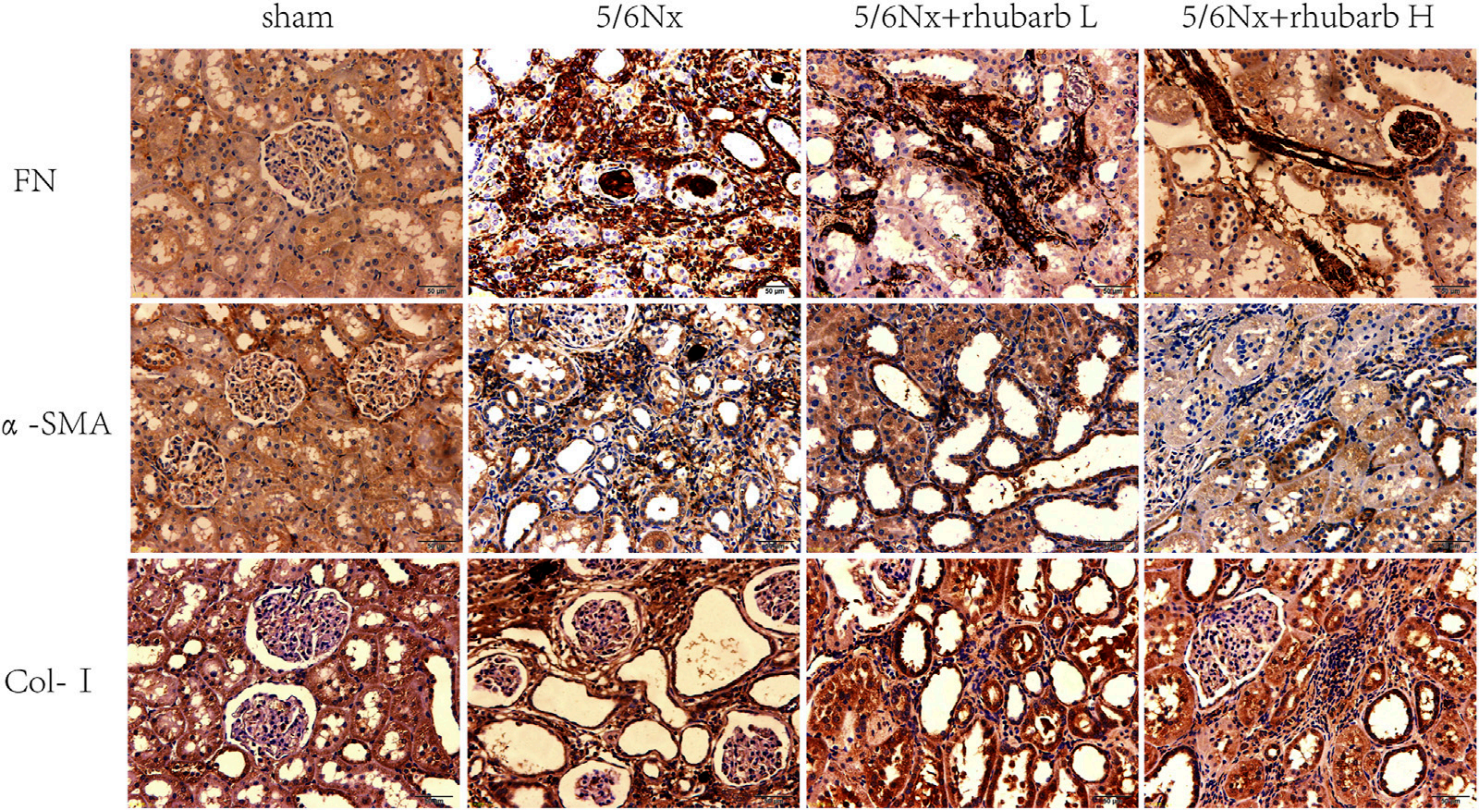
Immunohistochemistry. In 5/6Nx rats, fibrin-associated protein marks in kidney tissue of 5/6 nephrectomy rats were significantly increased, and rhubarb enema could reduce the expression of these markers (The brown-black area means the immunohistochemical positive area). FN, fibronectin. *α*-SMA, alpha smooth muscle actin. Col-I, collagen- I (400×).

Tubulointerstitial fibrosis (TIF) is the common final pathway for CKD progressing to end stage renal disease. Immunohistochemistry staining showed that the expression of TIF markers including fibronectin (FN), alpha smooth muscle actin (*α*-SMA), and collagen-I (COL-I) in the kidney tissue of 5/6Nx CKD rats were increased significantly, and markedly inhibited after rhubarb enema treatment (Figure 2).

### 3.4 Serum Inflammatory Factor Levels

**FIGURE 3:**
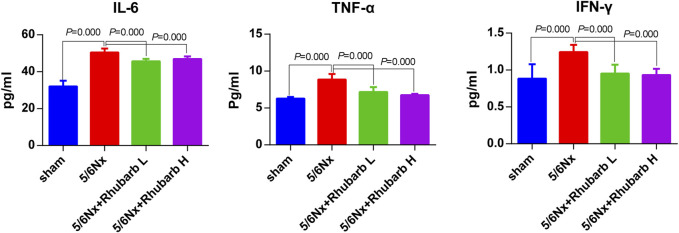
Serum inflammatory factors. In 5/6Nx rats, serum inflammatory factors in 5/6 nephrectomy rats were significantly increased, and reduced by rhubarb enema. IL-6, interleukin 6. TNF-α, Tumor necrosis factor alpha. IFN-γ, interferon gamma.

Compared with the control group, CKD rats have significantly higher levels of serum IL-6, TNFα and IFN-*γ*, which were significantly reduced after rhubarb enema intervention (Figure 3).

### 3.5 Serum TMAO and Its Precursor Levels

**FIGURE 4:**
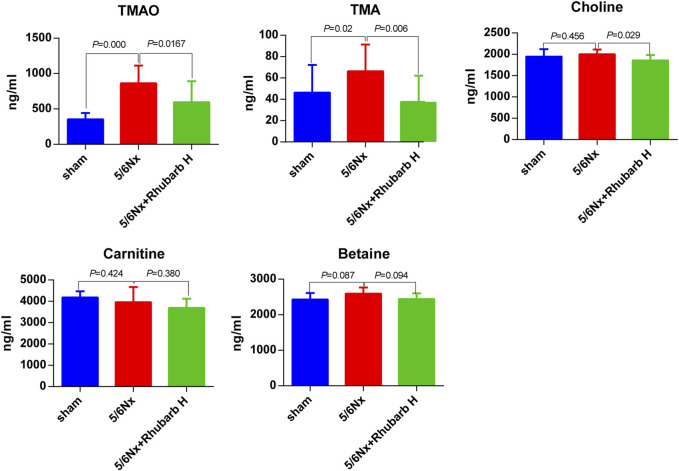
Serum TMAO and its precursors. Serum TMAO and TMA in 5/6 nephrectomy rats were markedly increased, but reduced significantly by rhubarb enema. TMAO, trimethylamine-N-oxide. TMA, trimethylamine.

The serum TMAO and TMA levels were elevated dramatically by 5/6Nx, compared with the control group (*p* < 0.001 for TMAO and *p* = 0.02 for TMA) and were reduced by high-dose rhubarb enema significantly (*p* = 0.0167 for TMAO and *p* = 0.006 for TMA). There was no significant difference of serum choline levels between the control group and the 5/6Nx model group, but still high-dose rhubarb enema reduced the serum choline level, with statistical significance (*p* = 0.029). Other precursors including betaine and carnitine did not show significant change among groups (Figure 4).

### 3.6 Rhubarb Enema Regulates Intestinal Flora

**FIGURE 5:**
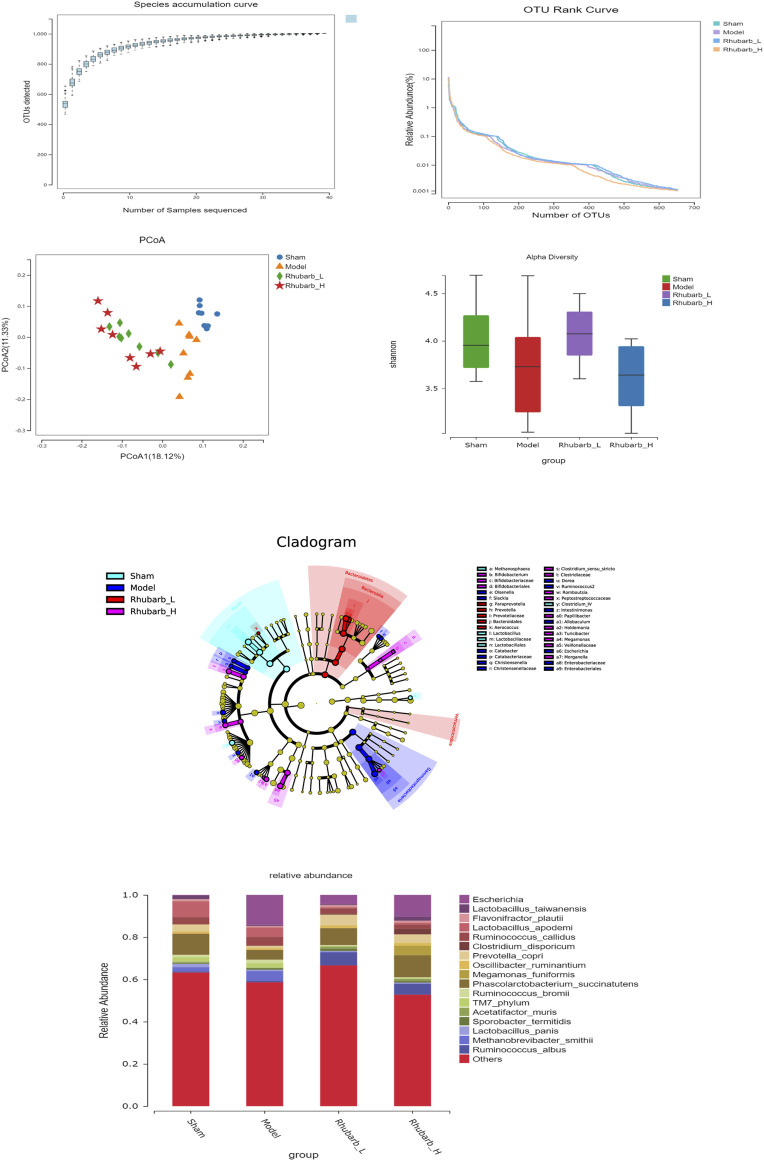
Characteristics of gut microbiota between groups. The abundance and structure of gut microbiota have been significantly changed in 5/6 Nx rats, and regulated by rhubarb enema.

**FIGURE 6:**
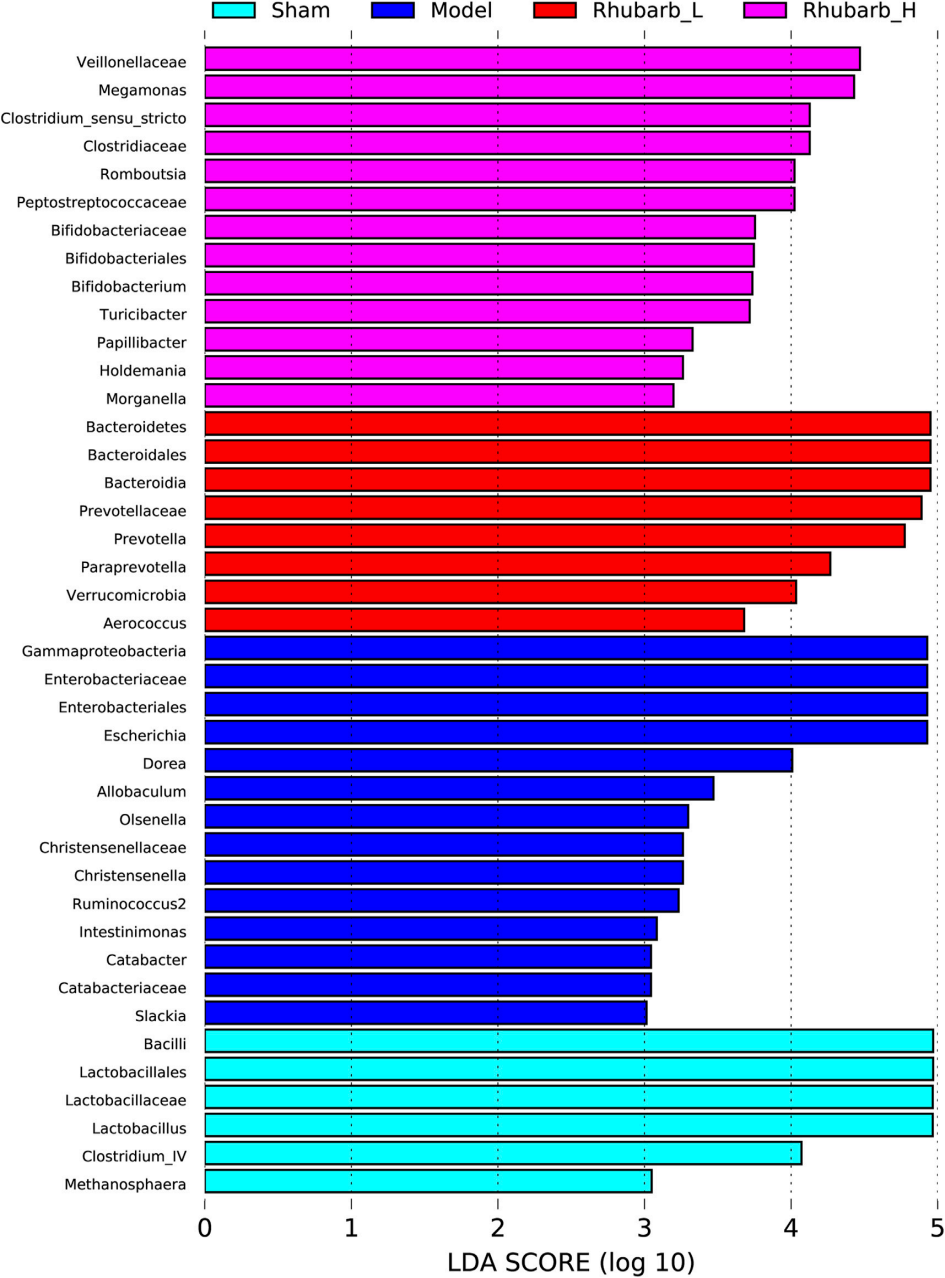
Differences in gut microbiota between groups at the genus level. Gut microbiota have been significantly changed in 5/6 Nx group and rhubarb enema groups.

The intestinal flora of the 5/6 Nx model rats was significantly more abundant and structural than the control group. For its variety, significant difference was also found between the 5/6 Nx model group and the rhubarb groups (Figure 5). Specifically, at the genus level, the differential flora enriched in the control group were *bacilli*, *lactobacilales, lactobacillaceae, lactobacillus, clotridium_IV, methanosphaera*. The differential flora enriched in the model group included *gammaproteobacteria, enterobacteriaceae, enterobacteriales, escherichia, dorea, allobaculum, olesenella, christensenellaceae, christensenella, ruminococcus2, intestinimonas, catabacter, catabacteriaceae,* and *slackia*. The differential intestinal flora enriched in the rhubarb groups (including low-dose and high-dose group) were *bacteroidetes, bacteroidales, bacteroidia, prevotella, prevotellaceae, paraprevotella, verrucomicrobia, aerococcus, veillonellaceae, megamonas, clostridium_sensu_stricto, clotostridiaceae, romboutcorepaceae, bifidobacteriaceae, bifidobacteriales, bifidobacterium, turicibacter, papillibacter, holdemania, morganella* (Figure 6)*.*

### 3.7 Function Prediction and Correlation Analysis of Intestinal Flora

We also performed functional predictions on the differential flora mentioned in 3.6. The results showed that the differential metabolic pathways of the intestinal flora between the control group and the model group were mainly apoptosis, amoebiasis, protein digestion and absorption, zeatin biosynthesis, nicotinate and nicotinamide metabolism, drug metabolism_other enzymes, penicillin and cephalosporin biosynthesis, phenylalanine metabolism, homologous recombination, peroxisome. The differential metabolic pathways of the intestinal flora between the 5/6Nx model group and the rhubarb groups (including low-dose and high-dose group) were mainly polycyclic aromatic hydrocarbon degradation, glycolysis/gluconeogenesis, other glycan degradation, apoptosis, synthesis and degradation of ketone bodies, glycine/serine and threonine metabolism, nitrotoluene degradation, protein digestion and absorption, plan-pathogen interaction, zeatin biosynthesis, propanoate metabolism, sulfur relay system, sphingolipid metabolism, drug metabolism-other enzyme, purine metabolism, ribonucleic acid (RNA) transport, Nucleotide-binding, oligomerization domain (NOD) like receptor signaling pathway, ribosome biogenesis in eukaryotes, proteasome, mRNA surveillance pathway, methane metabolism, basal transcription factors (Supplementary Data Sheets S1–3).

**FIGURE 7:**
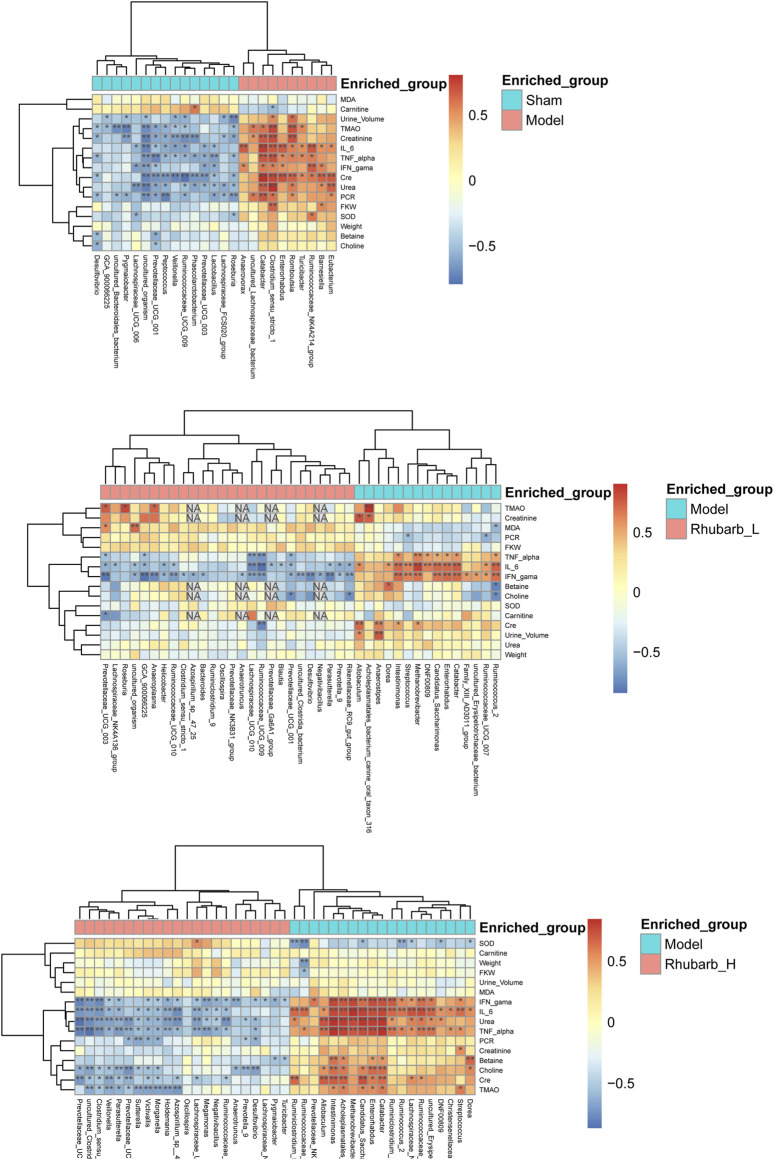
Correlation analysis. The correlation between all the differential intestinal flora and TMAO and its precursors, inflammatory factors, etc. (* Mean there was a significant correlation between the two interactive indicators. NA means the Missing value.).

In addition, we analyzed the correlation between all the differential intestinal flora and TMAO and its precursors, inflammatory factors, etc. The results showed that TMAO had negative correlation with *bacteroidales, lactobacillus, vellonella, roseburia, prevotella*, and *prevotellaceae*, but was positively correlated with *lachnospiraceae and romboutsia*. In terms of inflammatory factors, IL-6 was negatively correlated with *lachnospiraceae, bacteroides, rothia, lactobacillus, veillonella, ruminococcaceae, roseburia,* and positively correlated with *escherichia, eubacterium, romboutsia,* and *clostridium*. TNF-α was positively correlated with *clostrium, prevotellaceae, lachnospiraceae, ruminiclostridium,* and *methanobrevibacter*, and negatively correlated with *veillonella* and *lactobacillus*. IFN-*γ* was negatively correlated with *negativibacillus, prevotellaeae, ruminococcaceae,* and *parasutterella*, and positively correlated with *cadidatus, saccharimonas, intestinimonas, catabacter, enterorhabdus*, and *eubacterium* (Figure 7)*.*

## 4 Discussion

Our research preliminarily confirmed that serum TMAO levels in 5/6 Nx rats were significantly increased, and rhubarb enema could significantly reduce circulating TMAO in CKD rats, which may be related to the regulation of related intestinal flora. Carnitine, choline and betaine from red meat, eggs and dairy products degrade to TMA by related enzyme from some bateria. Then, TMA is oxidized in the liver by FMO3 into TMAO. Carnitine, choline are mainly found in foods of animal origin, such as meat (especially red meat), meat products, eggs and shellfish, while betaine is mainly found in plants (Jonsson and Backhed, 2017; Janeiro et al., 2018). TMAO is known to be closely related to cardiovascular disease and poor survival outcomes (Abbasi, 2019; Yang et al., 2019; Duttaroy, 2021). The mechanisms of this correlation has been well studied. TMAO stimulates the release of intracellular calcium ions and promotes platelet activation (Zhu et al., 2016; Yang et al., 2018). TMAO also affects cholesterol metabolism by affecting bile acid and apolipoprotein transporters, causing lipid metabolism disorders (Wang et al., 2011). Moreover, the impact of TMAO on kidneys has also been emphasized in recent years. TMAO excretion decreases after renal function declines since TMAO is excreted mainly through urine. TMAO accumulated in the kidney affects the abundant blood vessels in the kidney (Tang et al., 2015). Circulating TMAO promotes oxidative stress and inflammation in the kidney, contributing to renal interstitial fibrosis and dysfunction. TMAO has been shown to increase the production of pro-inflammatory cytokines, such as TNF-α, IL-1β and IL-6 (Sun et al., 2017). The present study also showed that the levels of inflammatory factors (IL-6, TNF-α, IFN-*γ*) in 5/6 Nx rats were significantly increased, and rhubarb enema significantly reduced the serum IL-6 and TNF-α.

Intestinal flora is an indispensable factor involved in the formation of TMA (Wang et al., 2011). The specific genus involved in the formation of TMAO are the choline-degrading bacterium *Desulfovibrio desulfuricans, Acinetobacter* and *Serratia*, *Gammaproteobacteria* (*Klebsiella pneumoniae, E. coli, Citrobacter, Providencia,* and *Shigella*), *Betaproteobacteria* (*Achromuteobacter*), *Firmic Actinobacteria*, they appear to be absent in *Bacteroidetes* (Craciun and Balskus, 2012; Falony et al., 2015; Zeisel and Warrier, 2016). In addition, TMA is involved in gastrointestinal methane production; in the human intestine, this pathway is in a *Methanomassiliicoccus* and *Methanomethylophilus*, as well as in *Methanobrevibacter smithii* and *Methanosphaera stadtmanae* (Falony et al., 2015). The present study shows that the genus related to TMAO were *intestinimonas*, *methanobrevibacter, parasutterella, anaerostipes, catabacter, intestinimonas, ruminiclostridium, desulfovibrio, clostridia,* and *parasutterella*. It can be seen that our research has both similarities and differences with other scholars’ studies. The reason for this difference may be that the different models have certain effects on the structure and composition of the intestinal flora, but still it suggests that 5/6 Nx CKD rats have increased TMAO-related flora, and rhubarb enema can inhibit those flora.

**FIGURE 8:**
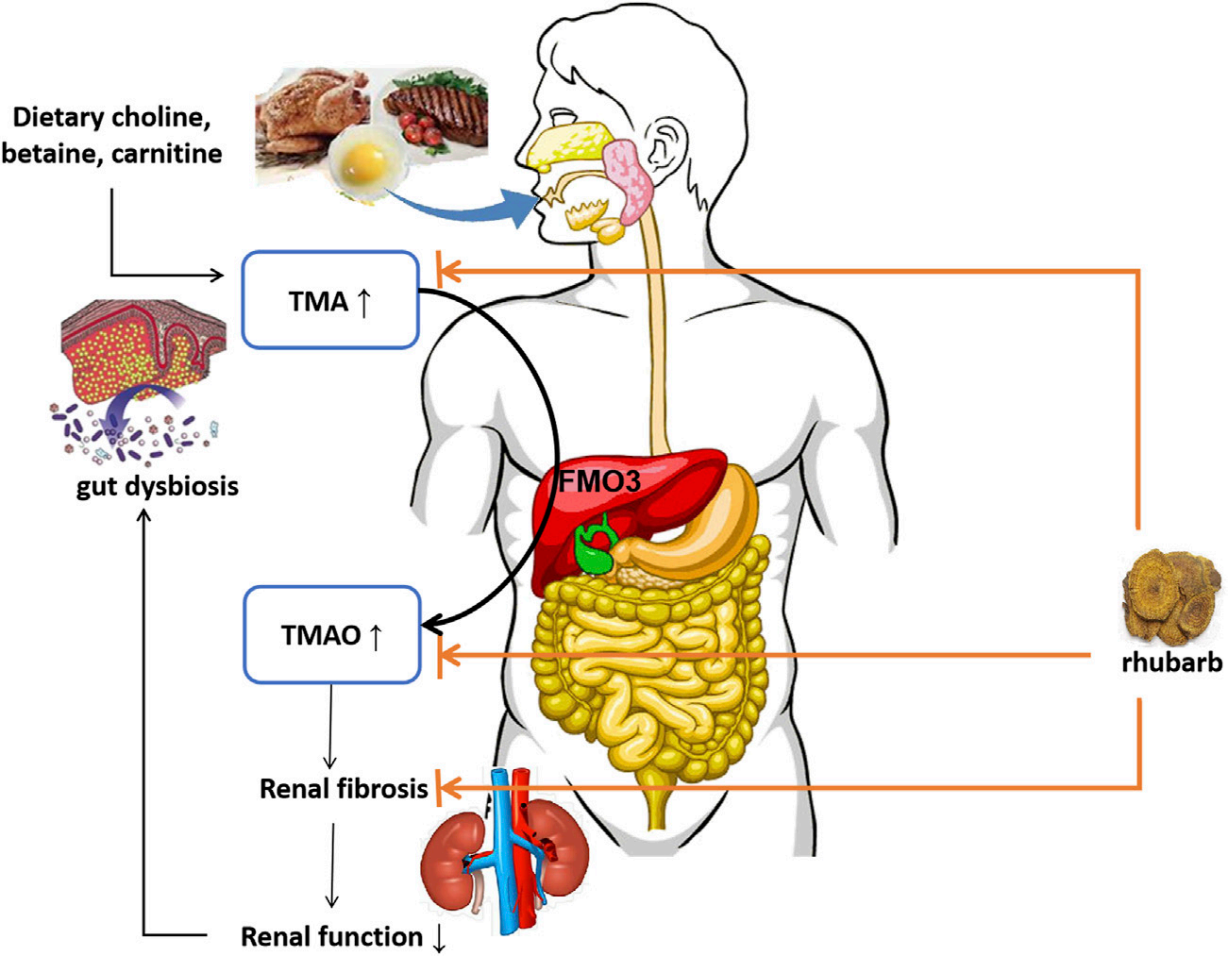
Schematic diagram. TMA: trimethylamine; FMO3: flavin-containing monooxygenase-3; TMAO: trimethylamine N-oxide.

TMAO accumulation has close relationship with progressive renal dysfunction and renal fibrosis. In animal models, elevated dietary choline or TMAO directly led to progressive renal tubulointerstitial fibrosis and dysfunction (Tang et al., 2015). TMAO accelerates the development of diabetic kidney disease (DKD) in a high-fat diet/low-dose streptozotocin-induced diabetes rat model. By feeding drinking water containing TMAO, the DKD rats showed exacerbated kidney dysfunction accompanied with renal fibrosis (Fang et al., 2021). A structural analog of choline, 3,3-dimethyl-1-butanol (DMB), was shown to reduce TMAO levels in mice fed by a high-choline or l-carnitine diet (Wang et al., 2011). Iodomethyl Choline (IMC), recently known as a non-lethal inhibitor of gut microbial TMA production, significantly reduced circulating TMAO level and multiple markers of renal injury including plasma creatinine, cystatin C and Fibroblast Growth Factor 23, attenuated development of microalbuminuria. Also, histopathologic evidence of renal fibrosis was reduced (Zhang et al., 2021). The mechanism of DMB and IMC lowering circulating TMAO level relies on non-lethally inhibiting TMA formation from microbes, inhibiting microbial TMA lyases, and shifting intestinal microbial communities. Previously our team had found that Emodin (one of typical active components of rhubarb) enema therapy regulated gut microbial communities toward a healthy status in 5/6 Nx rat model, it reduced the number of harmful bacteria, such as *Clostridium* spp., but augmented the number of beneficial bacteria, including *Lactobacillus spp* (Zeng et al., 2016). In the present study, we found rhubarb enema therapy reduced circulating TMA and TMAO levels, while it hardly affected choline, betaine or carnitine production. Meanwhile, the intestinal flora has changed. Thus, we speculate that rhubarb enema reduces circulating TMAO level by inhibiting TMA production through reducing involved bacteria in the gut. By targeting TMA/TMAO production, inflammation and renal fibrosis, rhubarb enema breaks the vicious circle on the gut-kidney axis. (Figure 8). To clarify the underlying mechanism, more experiments are needed.

Chinese herbal is commonly given by decoction in clinical practice, but for CKD which requires a long-term treatment, rhubarb administered by enema is preferred. On the one hand, rhubarb contains anthraquinone substances, long-term intragastric administration can cause diarrhea and melanosis *coli* or pseudo-melanoderma of colon (Zhu, 2004; Abendroth et al., 2009). Compared with intragastric administration, rhubarb administered by enema has shorter action time and smaller range of action, hence associated side effects are supposed to be avoided. On the other hand, enema is deemed a typical approach to deliver rhubarb to the colon, meanwhile it itself is a kind of lavage treatment. By its cleaning effect on the colon, rhubarb enema promotes the clearance of gut derived uremic toxin precursors, helping CKD patients excrete excessive toxins to some extent.

There are limitations of this study. One flaw is that there is no positive drug control group. On the one hand, this is not an accidental result, because the authors have found in previous works that rhubarb enema can significantly improve renal fibrosis and systemic inflammation in CKD rats, accompanied with decrease of blood creatinine levels, which may be related to the intestinal microbiota. (Lu et al., 2015; Zeng et al., 2016; Ji et al., 2020). On the other hand, we did not find a positive drug with the same mechanism of action as rhubarb enema. Another shortcoming of this study is that the relationship between TMAO and intestinal flora in this study was obtained based on bioinformatics analysis, which needs further verification.

The present work mainly found that rhubarb enema has a significant effect on reducing circulating TMAO level, and also reduces the levels of inflammatory factors and alleviates kidney fibrosis. In terms of intestinal flora, rhubarb enema reduces the genus of some bacteria associated with TMAO including *lachnospiraceae* and *romboutsia*. Therefore, we have explained to some extent that rhubarb enema may reduce the intestinal flora associated with TMAO production in 5/6 Nx rats, lower the serum TMAO level, thereby protecting from renal fibrosis and kidney function loss. Further verification of the TMAO-related intestinal flora involved in the study is also our next step. More work attempting to reveal the underlying mechanism of the intestinal-renal axis is warranted.

## 5 Conclusion

Rhubarb enema decreases circulating TMAO level and alleviates renal fibrosis in 5/6Nx CKD rats, which may be related to the regulation of intestinal microbial community.

## Data Availability

All datasets for this study are available. The Sequence Read Archive (SRA) data (accession number PRJNA763725) have been uploaded to the NCBI public repository. This data can be found here: https://www.ncbi.nlm.nih.gov/bioproject/PRJNA763725.
