# Supplementary figures and images for: Rhubarb Enema Decreases Circulating Trimethylamine N-Oxide Level and Improves Renal Fibrosis Accompanied With Gut Microbiota Change in Chronic Kidney Disease Rats
Source: Front Pharmacol. 2021 Dec 13;12:780924. doi: 10.3389/fphar.2021.780924 (PMC8710758; doi:10.3389/fphar.2021.780924)

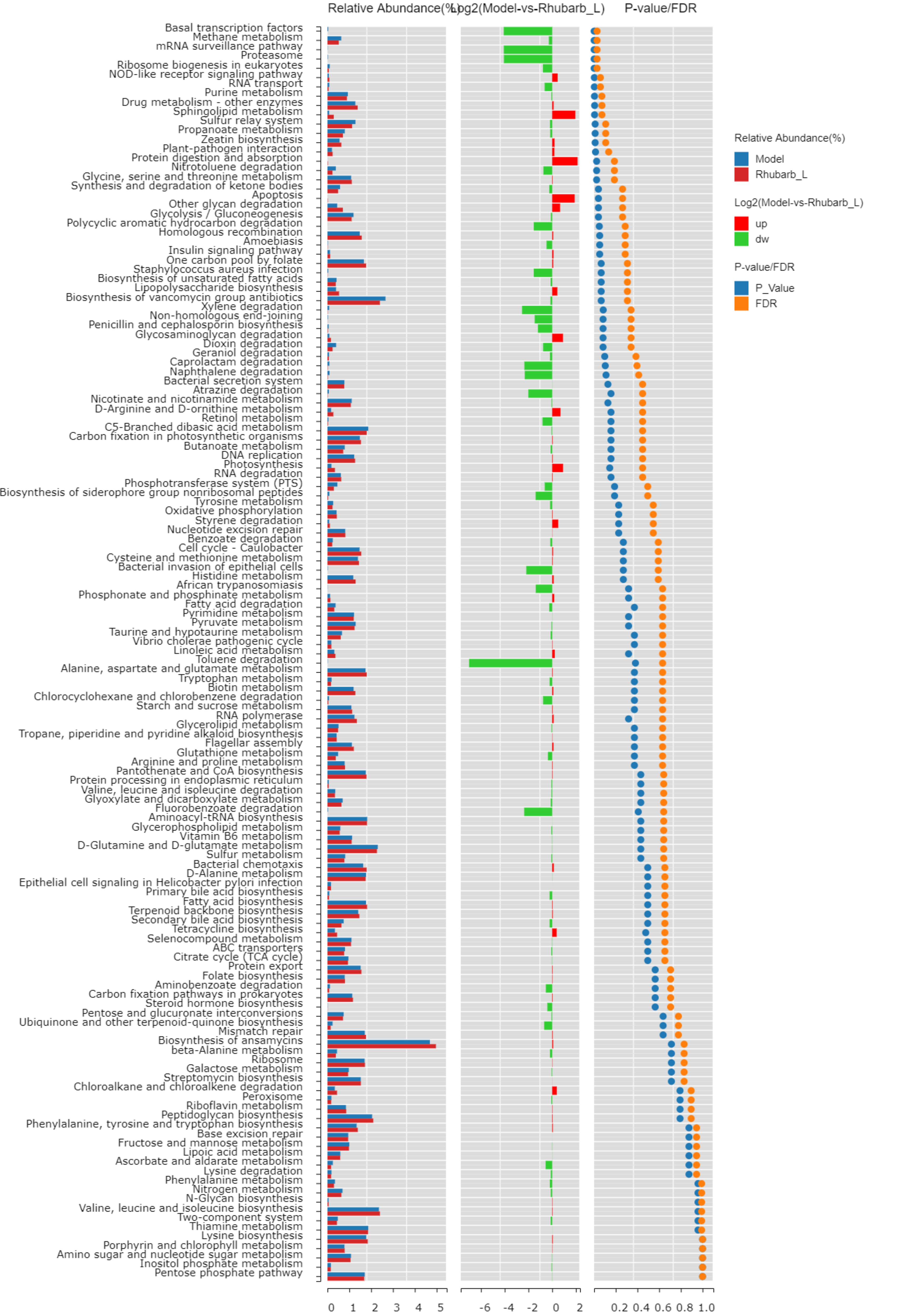

Supplement: Supplementary file 1 [file DataSheet2.PDF]

功能差异分析

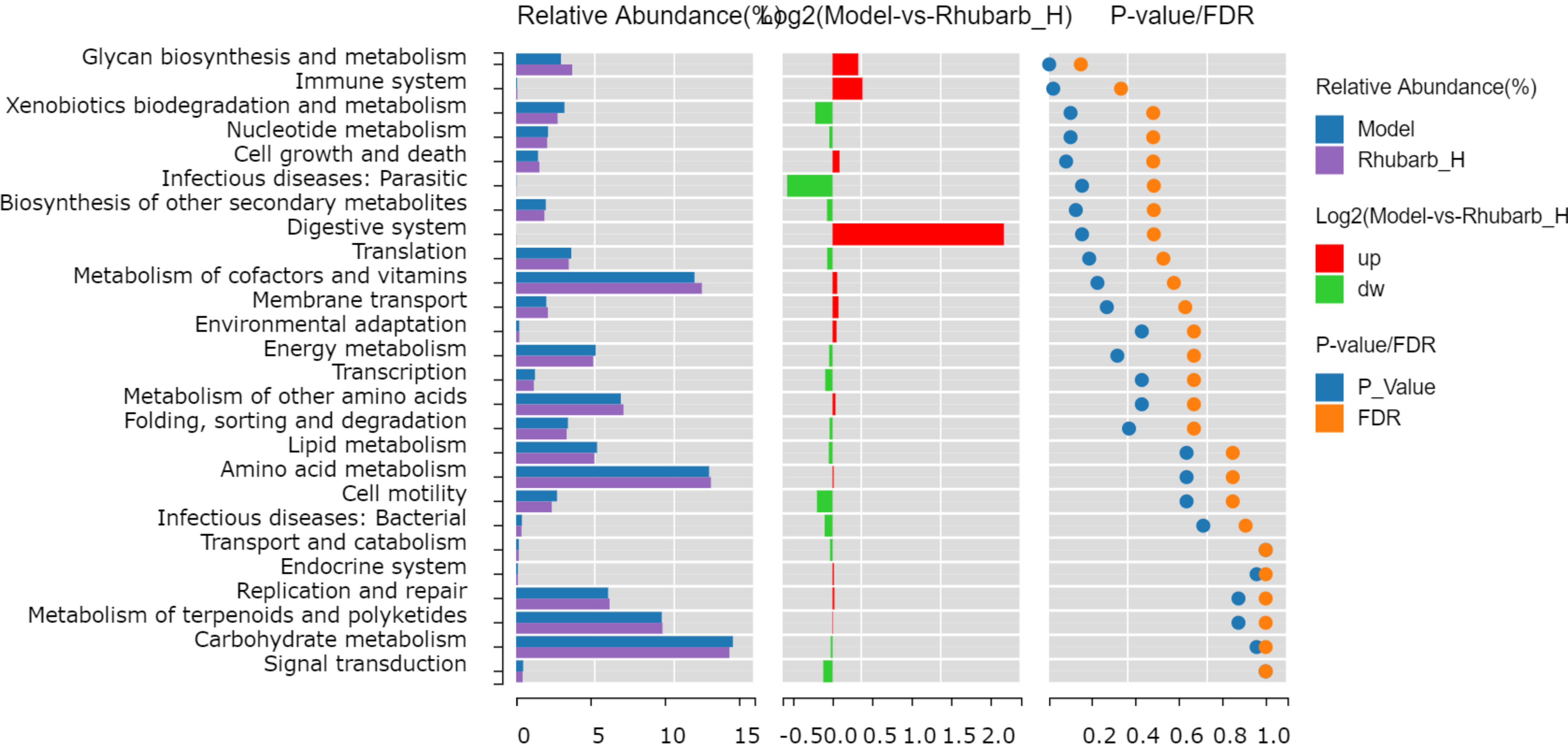

Supplement: Supplementary file 3 [file DataSheet3.PDF]

功能差异分析

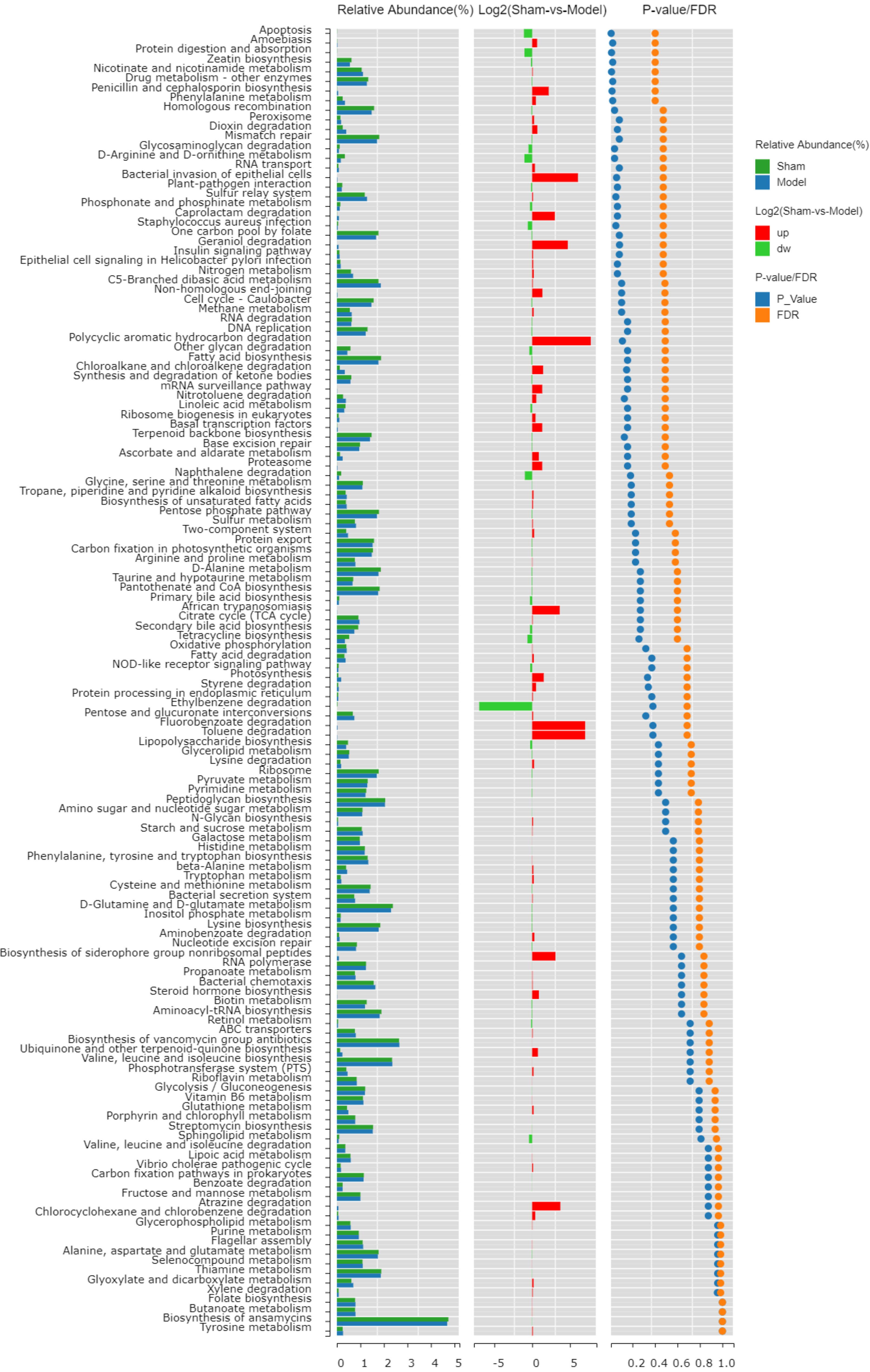

Supplement: Supplementary file 4 [file DataSheet1.PDF]
