# Supplementary material for: Rhubarb Enema Decreases Circulating Trimethylamine N-Oxide Level and Improves Renal Fibrosis Accompanied With Gut Microbiota Change in Chronic Kidney Disease Rats
Source: Front Pharmacol. 2021 Dec 13;12:780924. doi: 10.3389/fphar.2021.780924 (PMC8710758; doi:10.3389/fphar.2021.780924)
Supplement: Supplementary file 2 [file DataSheet4.docx]

**Raw data** for manuscript 780924 "Rhubarb enema decreases circulating Trimethylamine N-oxide level and improves renal fibrosis accompanied with gut microbiota change in CKD rats"

**H＆E (all microscopy images)** download links

Download link

https://www.jianguoyun.com/p/DSOny3IQiMPvCRiIvI8E (Access password: uyod8k)

**Immunohistochemistry（all microscopy images）**download links

download link

https://www.jianguoyun.com/p/DefYC48QiMPvCRiPvI8E (Access password: lc40cr)

**16S rDNA sequencing clean data**

download links:

https://www.jianguoyun.com/p/DSjZjzsQ9MPvCRi39pAE (Access password: 334fpq)

https://www.jianguoyun.com/p/DbbbhxQQ98PvCRihvI8E (Access password: wh4k7b)
